# The sex-specific difference in age-related aortic regional morphological changes

**DOI:** 10.1007/s40520-025-02981-1

**Published:** 2025-03-11

**Authors:** Zixuan Meng, Lele Cheng, Wenjun Liu, Yue Yu, Hui Liu, Guolin Yao, Jian Yang, Yue Wu, Zhijie Jian

**Affiliations:** 1 Department of Cardiovascular Medicine, The First Affiliated Hospital of Xi’an Jiaotong University, Xi’an, 710061 People’s Republic of China; 2 Department of Radiology, The First Affiliated Hospital of Xi’an Jiaotong University, Xi’an, 710061 People’s Republic of China; 3 Department of Radiology, Xi’an Gao Xin Hospital, Xi’an, 710075 People’s Republic of China; 4 Biobank, The First Affiliated Hospital of Xi’an Jiaotong University, Xi’an, 710061 People’s Republic of China

**Keywords:** Aorta aging, Arterial diameters, Arterial tortuosity, Sex disparities, Computed tomography

## Abstract

**Background:**

This study aims to investigate the influence of sex on age-related changes in aortic morphology using computed tomography (CT) imaging.

**Method:**

Patients who underwent contrast-enhanced chest and abdominal CT between July 2021 and April 2022 were enrolled and stratified into six groups. Sex-specific comparisons of body surface area (BSA)-adjusted aortic diameters and tortuosity were performed across different groups. To validate the consistency of sex differences on age-related arterial changes, particularly regarding stiffness, relevant data were extracted from a previously published study to analyze the correlation between age and arterial stiffness in both sexes.

**Results:**

A total of 208 participants (59.6% males, overall mean age: 60.13 ± 16.33 years) were enrolled. The BSA-adjusted diameters showed a positive correlation with age in both sexes, with females exhibiting a more rapid increase than males. In the age groups of 60–69 years and above 80 years, females had significantly larger BSA-adjusted diameters of the ascending aorta than males. Additionally, after 40 years old, females had significantly greater BSA-adjusted tortuosity in all aortic segments than males. Both aortic and descending thoracic aortic tortuosity exhibited a notable increase with aging, particularly in females. Furthermore, branchial-pulse wave velocity (baPWV) showed a similar age-related progression pattern, with baPWV increasing at an accelerated rate in females.

**Conclusion:**

A sex-based variation in the rate of aortic morphological aging is observed throughout the lifespan, with females showing more pronounced changes in aortic tortuosity. It highlights the importance of prioritizing preventive measures for females, who may benefit more due to sex-specific disparities.

**Supplementary Information:**

The online version contains supplementary material available at 10.1007/s40520-025-02981-1.

## Introduction

Aging is a significant risk factor for cardiovascular disease (CVD), particularly in relation to the aorta pathology [1]. There are apparent sex differences in susceptibility to aortic diseases in older people. While the prevalence of aortic aneurysm is higher in males, it demonstrates a more rapid progression and elevated mortality rate in older females [2]. Surgical outcomes for aortic dissection and aneurysms are generally less favorable in older females compared to males [3, 4]. Furthermore, older females have a higher incidence of heart failure with preserved ejection fraction, where aortic stiffness and increased cardiac afterload play key roles [5]. While females may initially seem to have a natural protection against CVD, the development of the condition in some older females suggests that their aortas may harbor more pronounced pathological changes or face greater significant hemodynamic challenges. These factors may ultimately lead to poorer vascular outcomes in females. Therefore, a better understanding of age-related sex differences in aortic pathophysiology can improve the targeted interventions and prevention strategies.

The aging process induces characteristic structural modifications in aortic morphology, such as tortuosity and dilation. These morphological changes can impact aortic hemodynamics, and increase cardiac afterload, resulting in aortic wall over-stretching, all of which are well-established contributors to the pathogenesis of atherosclerosis and CVD [6–8]. Advanced imaging modalities, particularly contrast-enhanced computed tomography (CT) and magnetic resonance imaging (MRI), have enabled precise quantification of age-related anatomical changes in the aorta, revealing significant sex-specific variations in aortic diameters and curvature [9–11]. A cross-sectional study from the Multi-Ethnic Study of Atherosclerosis (MESA) has reported a gradual age-dependent increase in ascending thoracic aortic diameter in both sexes by MRI, while after adjusting for body surface area (BSA), females consistently exhibited greater aortic diameters than males [11]. The investigation of descending aortic tortuosity using enhanced CT also revealed a more pronounced age-associated increase in females than in males [12]. However, these studies have only focused on a certain aortic segment, examining either aortic diameter or tortuosity in isolation, without a comprehensive evaluation of the entire aortic morphology. Consequently, the sex-specific patterns of global aortic morphological changes throughout the aging process remain poorly characterized, necessitating systematic investigations to elucidate sex differences in aortic remodeling throughout the lifespan.

The present study was designed to comprehensively evaluate sex-specific differences in age-related aortic morphological changes using contrast-enhanced CT imaging to quantify both diameters and tortuosity across multiple aortic segments.

## Methods

### Study population and data collection

A cross-sectional study was conducted at the First Affiliated Hospital of Xi’an Jiaotong University, enrolling patients who underwent chest and abdomen contrast CT imaging between July 2021 and April 2022. The exclusion criteria included: (1) a prior diagnosis of CVD, hypertension, diabetes mellitus, or chronic kidney disease; (2) the presence of aneurysms, dissection, vascular malformation, or other vascular variations on CT scans; (3) Inadequate quality CT scans with significant artifacts; (4) incomplete clinical data for comprehensive analysis. Demographic and clinical data were extracted from the medical records, including age, sex, weight, height, body mass index (BMI), systolic blood pressure (SBP), and diastolic blood pressure (DBP). Current smoking was defined as positive smoking. Related laboratory data including white blood cell counts, cholesterol, albumin, serum glucose, urea, creatinine, uric acid, and estimated glomerular filtration rate (eGFR) were assayed by an automated chemistry analyzer. All the laboratory tests were conducted within 48 h before CT scans. Written informed consents were obtained from all participants, and this study was approved by the ethics committee approval of the First Affiliated Hospital of Xi’an Jiaotong University (Approved number: XJTU1AF2020LSL-018).

### Assessment of aortic diameters and tortuosity index calculation

**Fig. 1:**
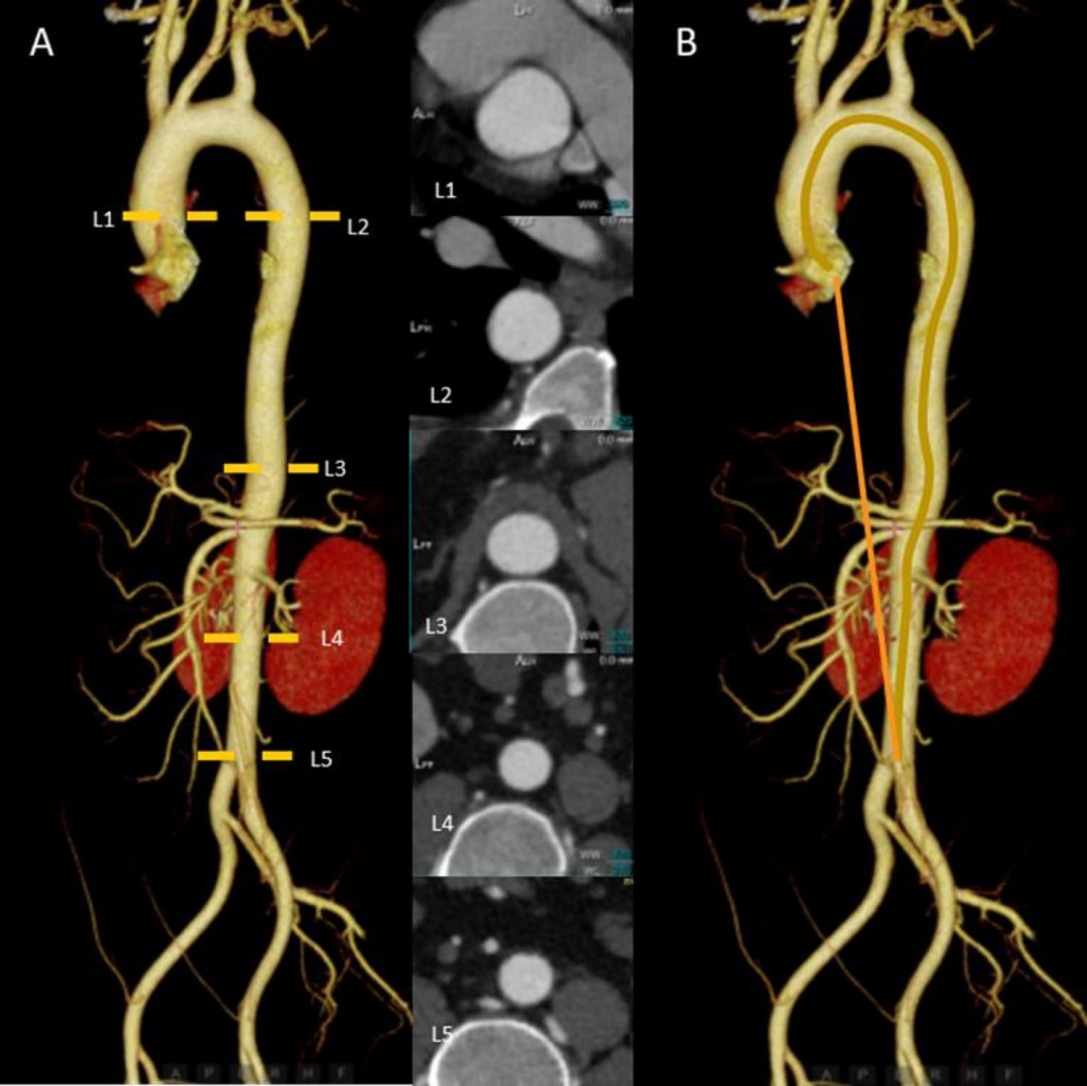
The measurements of aortic morphology. **A** Sites of diameter measurements of the aorta; **B** Measurement of tortuosity index. The yellow line is the true length measurement of the vessel, and the orange line is the straight-line length. The tortuosity index of the aorta is the true length / the straight-line length (colour figure online)

Two 256-slice CT scanners (Philips Brilliance iCT from Medical Systems in Best, Netherlands, and the Revolution CT from GE Healthcare in Milwaukee, WI) were utilized to conduct a thoracoabdominal enhanced CT scan. The scan encompassed the area from the lung apices to the iliac crest and was performed without electrocardiograph (ECG) gating. All imaging data were analyzed using a standard post-processing workstation (uInnovation-CT, R001, United Imaging Healthcare, Shanghai, China) equipped with commercially available volume viewer software. The aortic diameters were measured at five predefined anatomical levels using the outer edge-to-outer edge method: (1) L1: the 1 cm distal to the sinotubular junction on the ascending aorta; (2) L2: at the same axial level as L1 on the descending aorta; (3) L3: the intersection of aorta and diaphragm; (4) L4: the midpoint between L3 and L5; (5) L5: 3 cm above the level of the aortic bifurcation (Fig. 1A).

A proficient radiologist specializing in cardiovascular imaging accurately identified the aorta of the corresponding segment by pinpointing two points on the corresponding anatomical landmark, then we calculated the tortuosity of the whole aorta, thoracic aorta, and abdominal aorta separately [13]. For aortic tortuosity assessment, the aortic length was defined as extending from the aortic valve plane to the iliac bifurcation. An automated centerline reconstruction algorithm was then applied between these two reference points (Fig. 1B). The tortuosity index of the aorta was then calculated by dividing the length of the centerline by the straight-line length. The detailed methodology for these measurements has been previously described in our published work [14]. Meanwhile, we standardized the aortic diameters and tortuosity by BSA to exclude the effect of diverse body shapes on the data analysis. BSA was calculated by the Dubois formula which was referred to as BSA (m^2^) = 0.007184 × [height (cm)^(0.725)^] × [weight (kg)^(0.425)^] [15].

### Reproducibility analysis

In order to ensure the reproducibility of morphological measurements, 30 patients were randomly selected, and conducted duplicate measurements of aortic diameters and tortuosity by two independent investigators. Intra-class correlation coefficient (ICC) and Bland–Altman plot were used to evaluate inter-observer consistency to ensure measurement reliability and analytical accuracy.

### Statistical analysis

Patients enrolled were divided into six age groups: < 40, 40 ~ 49, 50 ~ 59, 60 ~ 69, 70 ~ 79, and ≥ 80 years. All the continuous data in accordance with normality distribution were presented as mean ± SD and compared by t-test. For non-normally distributed continuous variables, the Kruskal–Wallis test was used for group comparisons. The categorical variables were presented as numbers and percentages and compared by χ^2^ test. Multiple linear regression analysis was performed to analyze the potential factors associated with aortic aging. Sex-specific associations between BSA-adjusted aortic dimensions and aging were evaluated using linear regression models. A *p*-value < 0.05 was regarded as statistically significant for all tests. All statistical analyses were performed by R software (version 4.0.2).

## Results

### Characteristics of participants

A total of 208 participants were enrolled in this study. The overall average age was 60.13 ± 16.33 years old, and 59.6% of participants were males. The overall mean BSA was 1.71 ± 0.19 m^2^. No significant age difference was observed between sexes (females: 60.06 ± 16.97 years; males: 60.18 ± 15.95 years; *p* = 0.959). Blood pressure, white blood cells, cholesterol, albumin, glucose, urea, creatinine, uric acid, and eGFR also showed no significant sex-based differences. On the contrary, males exhibited significantly higher levels of BMI and BSA compared to females (*p* = 0.019 and *p* < 0.001 respectively). The proportion of male smokers was also significantly higher than that of female smokers (*p* < 0.001). The clinical characteristics are summarized in Table 1.

**Table 1:** Characteristics of participants according to sex

Variables	All participants (n = 208)	Male (n = 124, 59.6%)	Female (n = 84, 40.4%)	*p* value
Age, years	60.13 ± 16.33	60.18 ± 15.95	60.06 ± 16.97	0.959
BMI, kg/m^2^	23.07 ± 4.03	23.61 ± 4.11	22.27 ± 3.80	0.019
BSA, m^2^	1.71 ± 0.19	1.79 ± 0.16	1.57 ± 0.16	< 0.001
Current smoking, n (%)	76 (36.5%)	75 (60.5%)	1 (1.2%)	< 0.001
SBP, mmHg	124.12 ± 17.33	124.89 ± 16.65	123.83 ± 18.73	0.549
DBP, mmHg	75.55 ± 9.23	76.53 ± 9.20	74.81 ± 8.79	0.058
White blood cells, 10^9^/L	6.39 ± 2.82	6.08 ± 2.63	6.88 ± 2.92	0.058
Cholesterol, mmol/L	4.14 ± 1.51	4.17 ± 1.20	4.10 ± 1.91	0.203
Albumin, g/L	36.27 ± 5.42	36.80 ± 5.13	35.48 ± 5.78	0.087
Glucose, mmol/L	5.52 ± 2.12	5.60 ± 1.88	5.39 ± 2.45	0.132
Urea, mmol/L	5.48 ± 1.91	5.70 ± 2.00	5.18 ± 1.71	0.190
Creatinine, μmol/L	59.42 ± 18.86	61.37 ± 20.65	57.54 ± 16.67	0.086
Uric acid, μmol/L	285.54 ± 96.09	290.42 ± 94.55	280.53 ± 96.69	0.154
eGFR, ml/min/1.73m^2^	102.73 ± 20.36	102.87 ± 20.68	100.85 ± 21.90	0.623

*BMI* body mass index, *BSA* body surface area, *SBP* systolic blood pressure, *DBP* diastolic blood pressure, *eGFR* estimated glomerular filtration rate

### Aortic diameters increased more with age in females than in males

**Fig. 2:**
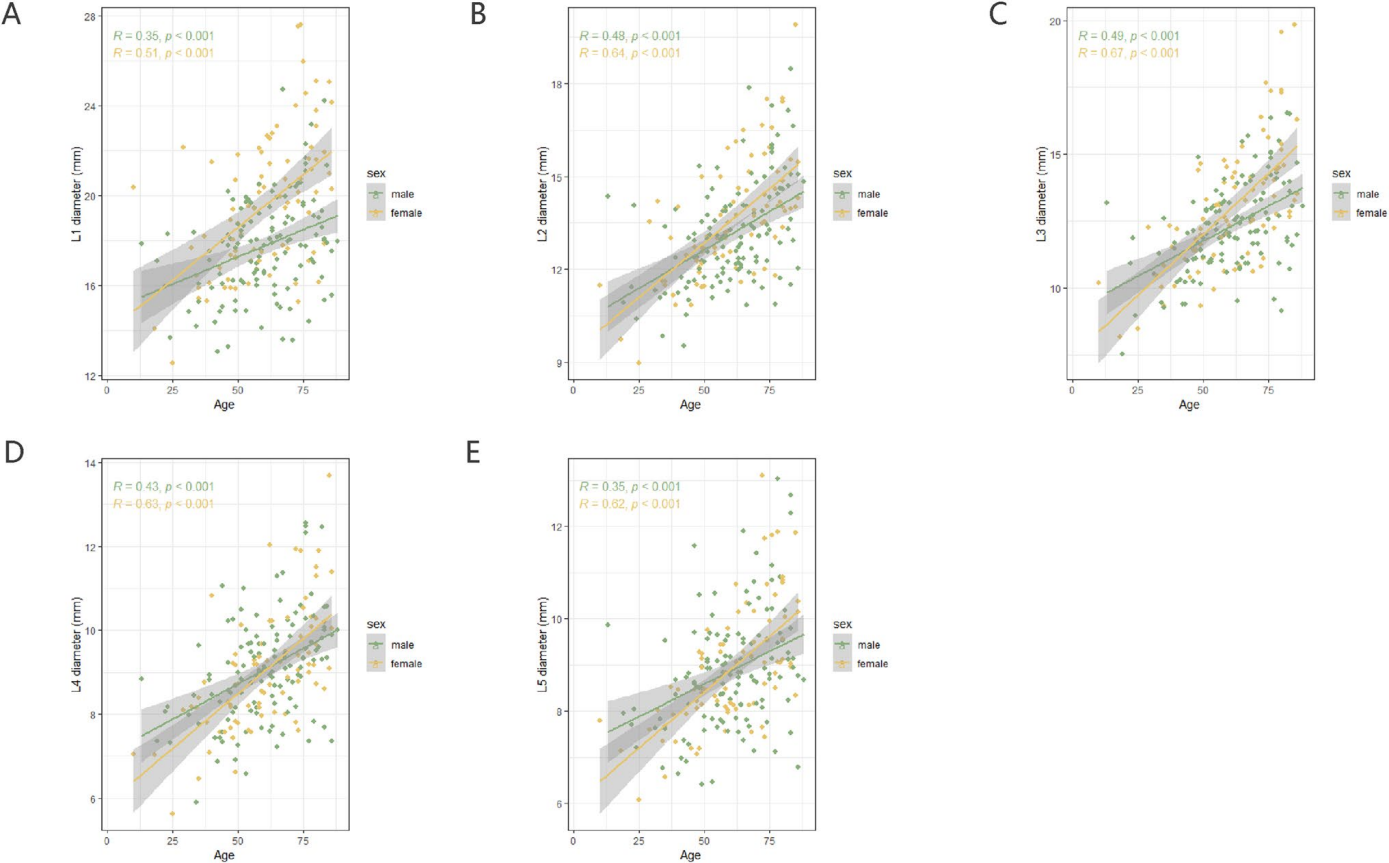
The relationship between BSA-corrected aortic diameters and age according to sex. Linear regression analysis showed a linear relationship between age and L1 (**A**), L2 (**B**), L3 (**C**), L4 (**D**), and L5 (**E**) in males and females

BSA-adjusted aortic diameters at all different measured levels demonstrated a positive correlation with age in both sexes, indicating that females exhibited a more repaid increase in aortic diameters with aging throughout the life course compared to males (Fig. 2).

**Fig. 3:**
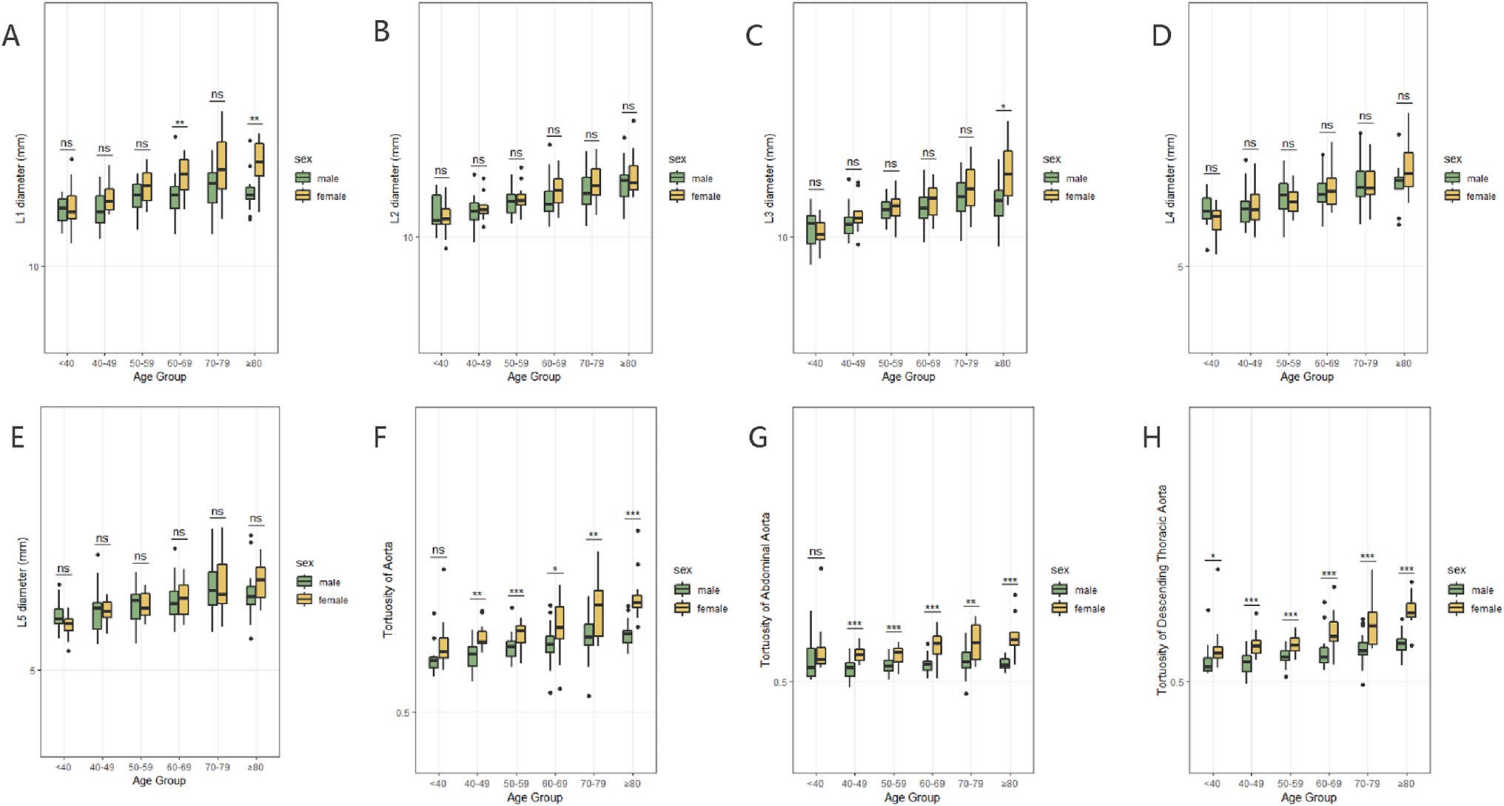
The boxplot shows the sex disparities in BSA-corrected aortic diameters and BSA-corrected aortic tortuosity in different age groups. At the age of 60 to 69 and above 80, L1 was significantly greater in females than in males (**A**). The L3 of females over 80 years old is larger than that of males (**C**). L2, L4, and L5 shows no sexual disparities in all ages (**B**, **D**, **E**). A significant sex difference in the tortuosity of the aorta and abdominal aorta appeared in people over 40 years of age (**F**, **G**). The tortuosity of descending thoracic aorta is greater in females than that of males within all age groups (**H**)

However, at the ages of 60–69 and above 80 years old, L1 was significantly larger in females than males. Similarly, the L3 in females exceeded that of males in the > 80 years age group, whereas there were no sexual disparities observed in L2, L4, and L5 across all age groups (Fig. 3, Supplementary Table 1).

### More remarkable age-related increase of segmental aortic tortuosity in females

**Fig. 4:**
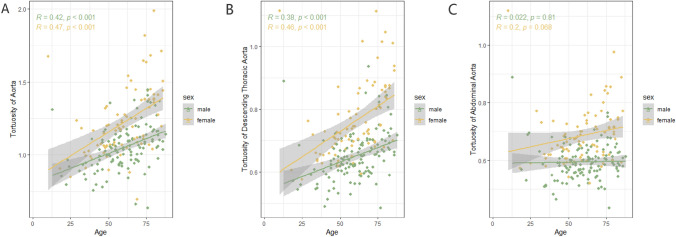
The relationship between BSA-corrected aortic tortuosity and age according to sex. Linear regression analysis showed a linear relationship between age and aortic tortuosity (**A**), and descending thoracic aortic tortuosity (**B**) in different sexes. A non-linear relationship existed in abdominal aortic tortuosity either in males or females (**C**)

Analysis of BSA-indexed aortic tortuosity revealed significant sex differences across most age groups (Supplementary Table 2). Among all age groups, females exhibited greater tortuosity in the descending thoracic aorta than males. Yet this sex distribution in the whole aorta and abdominal aorta became apparent in individuals aged over 40 years (Fig. 3). As individuals aged, there was a noticeable increase in the tortuosity of the whole aorta and descending thoracic aorta, especially in females. However, no significant linear correlation between abdominal aortic tortuosity and age was observed in either males or females (Fig. 4).

### Intra-observer and interobserver variability

The study demonstrated excellent intra-observer reliability for L1-L5, aortic tortuosity, and descending thoracic aortic tortuosity measurements, with ICC ranging from 0.926 to 0.994. Abdominal aortic tortuosity measurements also showed good reliability (ICC = 0.875). Regarding interobserver reliability, excellent agreement was observed for L2-L4 and descending thoracic aortic tortuosity (ICCs: 0.920–0.923), while L1, L5, aortic tortuosity, and abdominal tortuosity measurements demonstrated good reliability (ICCs: 0.847–0.894; Supplementary Table 4). Bland–Altman analysis revealed that all measurement differences between repeated assessments fell within the predetermined limits of agreement (Supplementary Figure S1 and S2).

### Based on BMJ open datasets: more remarkable age-related changes of arterial morphology in females

**Fig. 5:**
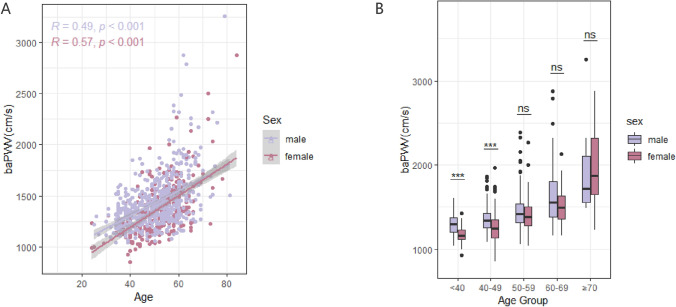
The relationship between baPWV and age according to sex based on a previously published article in BMJ Open (**A**). Sex disparities in baPWV in different age groups (**B**)

We also analyzed the sex-specific association between baPWV and aging for better understanding the characteristics of aortic aging. Data from a previously published paper recruited 912 patients (592 males and 320 females) aged 24–84 years old (Supplementary Table 3). Females were older than males, with higher levels of cholesterol and eGFR. Conversely, males presented higher blood pressure, glucose, and triglycerides than females, as well as the proportion of smokers. No significant difference existed in the baPWV of different sexes (males: 1411.5 cm/s, females 1363.0 cm/s). The association between age and baPWV was similar to aortic tortuosity. baPWV increased with age in both males and females, but the increase was more pronounced in females. Although males initially showed higher baPWV, females eventually surpassed them as they aged (Fig. 5).

## Discussion

Vascular morphological changes induced by aging are an inevitable and progressive process. Age-related changes in the arteries have a significant impact on cardiovascular remodeling and systemic organ function. As posited by the physician Thomas Sydenham in the seventeenth century, ‘a man is as old as his arteries’ [16]. Our study revealed a sexual dimorphism in age-related morphological changes of the whole aorta, with females experiencing more significant arterial remodeling, particularly in tortuosity, compared to males. This suggested that aortic aging manifested more prominently in females.

The observed sexual dimorphism in aortic tortuosity and dilation is not coincidental. Previous studies by Coutinho and Dart et al. have established that females were more susceptible to increased pulse pressure and arterial stiffness as they age [17, 18]. Another research by Cheng et al. demonstrated a sex-specific pattern of left ventricular (LV) wall thickness with age, and the increase in LV wall thickness was more pronounced in women after adjusting for body size [19, 20]. These structural changes are accompanied by functional alterations, as evidenced by more prominent age-related increases in LV ejection fraction and diastolic dysfunction in females [21, 22]. Our findings regarding aortic morphological changes provided a potential mechanistic explanation for these sex-specific cardiovascular adaptations.

Our study found that aortic diameters and tortuosity increased with age in both sexes, which is consistent with previous research [11]. Unlike other studies that focused on specific segments of the aorta [23, 24], we provided a comprehensive overview of age-related changes in aortic morphology. Specifically, there was a distinction in thoracic aortic diameter over 60 years old, with females having a larger value than males. However, sex difference in abdominal aortic diameter across all age groups was unapparent. At the age of above 40 years old, the tortuosity of all aortic segments was significantly greater in females. These findings indicated that sexual dimorphism in aortic aging primarily manifested through tortuosity rather than diameter changes, and females showed more significant arterial remodeling in the longitudinal direction compared to males over their lifespan. The increased tortuosity of the aorta results in alterations of hemodynamics, leading to higher turbulence, pressure, and wall shear stress [6, 25]. Our baPWV analysis further supported these observations, showing that while young and middle-aged males exhibited lower aortic tortuosity and higher arterial stiffness, females experienced accelerated progression of aortic lesions with aging, potentially explaining the higher incidence of aortic dissections in younger males, and accelerated progression of aortic lesions with poorer outcomes in older females [2, 26].

Our study also revealed significant age-related increases in all regional aortic diameters (correlation coefficients: 0.622–0.67), with more rapid progression in females. Notably, the most pronounced changes occurred in the descending thoracic to abdominal aorta. While whole aortic and descending thoracic aortic tortuosity showed significant age-related changes, abdominal aortic tortuosity remained stable. These findings emphasized the importance of considering regional variations in aortic aging studies, and that descending aortic morphology may be particularly informative for assessing aortic aging.

The observed sexual dimorphism in aortic aging, particularly in middle-aged and older females, suggested a sex-specific effect on aortic aging. While this observational study didn’t establish the precise mechanism, it was reasonable to speculate that estrogen plays a crucial role, especially considering the accelerated changes post-menopause. Estrogen can protect cardiovascular health by inhibiting the accumulation of collagen and the proliferation of vascular smooth muscle cells (VSMCs) [27]. The decline in estrogen levels may contribute to increased collagen deposition, aortic stiffness, and tortuosity. Additionally, estrogen-related bone and muscle loss may exacerbate arterial tortuosity through height reduction [14, 28, 29]. To elucidate the specific mechanisms, further research is warranted.

There are three main limitations in our study. Firstly, the data was extracted from a small-scale cross-sectional study conducted at a single center, unavoidable confounding factors may influence the outcome, potentially limiting the generalizability of our results. Secondly, non-ECG gating contrast CT was used to measure aortic diameters and tortuosity, which may affect measurement precision due to cardiac cycle variations. Inevitably, some of our subjects had malignant tumors, and we avoided CVD as much as possible through imaging examination and clinical diagnosis. To validate our findings and illustrate the underlying mechanisms, it is imperative to conduct larger-scale surveys with multi-center involvement and high-precision measurements of the normal population.

## Conclusion

The age-related morphological changes in aorta, particularly the tortuosity of the thoracic aorta, are more pronounced in females than males. These findings underscore the critical need for targeted prevention strategies to mitigate age-related aortic remodeling and its associated cardiovascular risks. Given the observed sexual dimorphism, prevention programs should prioritize female populations, who demonstrate greater susceptibility to these changes and may derive substantial benefit from early intervention and preventive measures.

## Supplementary Information

Below is the link to the electronic supplementary material.

Supplementary file1 (DOCX 26 KB)

Supplementary file2 (PPTX 506 KB)

## Data Availability

The datasets analyzed during the current study are available from the corresponding author on reasonable request.

## Declarations

### Reproduction permission

Reproduced with permission from BMJ open.
