# Supplementary material for: The sex-specific difference in age-related aortic regional morphological changes
Source: Aging Clin Exp Res. 2025 Mar 11;37(1):76. doi: 10.1007/s40520-025-02981-1 (PMC11897083; doi:10.1007/s40520-025-02981-1)
Supplement: Supplementary file 1 — Supplementary file1 (DOCX 26 KB) [file 40520_2025_2981_MOESM1_ESM.docx]

**Supplementary Table 1 Sex disparities in BSA-adjusted aortic diameters among different age groups.**

| BSA-adjusted aortic diameters (mm/m^2^) | Age groups | Male | Female | *p* value |
| --- | --- | --- | --- | --- |
| L1 | <40 | 16.35±1.60 | 16.73±2.74 | 0.928 |
|  | 40-49 | 16.40±2.06 | 17.75±1.79 | 0.116 |
|  | 50-59 | 17.86±1.62 | 19.08±1.99 | 0.069 |
|  | 60-69 | 17.94±2.17 | 20.23±2.16 | 0.003 |
|  | 70-79 | 18.85±2.52 | 21.28±3.87 | 0.054 |
|  | ≥80 | 18.39±2.27 | 21.68±2.80 | 0.007 |
| L2 | <40 | 12.03±1.55 | 11.61±1.54 | 0.833 |
|  | 40-49 | 12.12±1.28 | 12.42±0.28 | 0.561 |
|  | 50-59 | 12.85±1.07 | 13.28±1.04 | 0.281 |
|  | 60-69 | 13.15±1.48 | 13.96±1.53 | 0.095 |
|  | 70-79 | 13.90±1.69 | 14.54±1.64 | 0.267 |
|  | ≥80 | 14.66±1.93 | 15.36±1.98 | 0.462 |
| L3 | <40 | 10.72±1.64 | 10.32±1.37 | 0.487 |
|  | 40-49 | 11.13±1.35 | 11.74±1.41 | 0.125 |
|  | 50-59 | 12.24±0.93 | 12.51±1.35 | 0.377 |
|  | 60-69 | 12.44±1.47 | 12.93±1.54 | 0.384 |
|  | 70-79 | 13.36±1.64 | 14.15±2.05 | 0.292 |
|  | ≥80 | 13.01±2.07 | 15.65±2.48 | 0.014 |
| L4 | <40 | 8.10±0.97 | 7.51±0.94 | 0.134 |
|  | 40-49 | 8.38±1.17 | 8.33±1.06 | 0.955 |
|  | 50-59 | 8.97±1.02 | 8.67±0.77 | 0.257 |
|  | 60-69 | 9.13±1.05 | 9.28±1.10 | 0.811 |
|  | 70-79 | 9.74±1.36 | 9.68±1.23 | 0.989 |
|  | ≥80 | 9.73±1.22 | 10.53±1.49 | 0.193 |
| L5 | <40 | 8.10±0.90 | 7.46±0.68 | 0.091 |
|  | 40-49 | 8.38±1.34 | 8.26±0.74 | 0.750 |
|  | 50-59 | 8.71±0.92 | 8.64±0.71 | 0.779 |
|  | 60-69 | 8.88±1.10 | 8.99±1.02 | 0.772 |
|  | 70-79 | 9.57±1.37 | 9.84±1.64 | 0.692 |
|  | ≥80 | 9.35±1.59 | 10.00±1.02 | 0.145 |

**Supplementary Table 2 Sex disparities in BSA-adjusted tortuosity index among different age groups.**

| BSA-adjusted tortuosity index | Age groups | Male | Female | *p* value |
| --- | --- | --- | --- | --- |
| The tortuosity of aorta | <40 | 0.94±0.14 | 1.06±0.23 | 0.069 |
|  | 40-49 | 0.96±0.13 | 1.12±0.10 | 0.002 |
|  | 50-59 | 1.03±0.10 | 1.13±0.10 | ＜0.0001 |
|  | 60-69 | 1.06±0.14 | 1.20±0.23 | 0.011 |
|  | 70-79 | 1.13±0.19 | 1.35±0.25 | 0.009 |
|  | ≥80 | 1.15±0.11 | 1.45±0.21 | ＜0.0001 |
| The tortuosity of DTA | <40 | 0.62±0.10 | 0.69±0.15 | 0.027 |
|  | 40-49 | 0.60±0.06 | 0.70±0.07 | ＜0.0001 |
|  | 50-59 | 0.64±0.05 | 0.70±0.05 | ＜0.0001 |
|  | 60-69 | 0.65±0.08 | 0.77±0.10 | ＜0.0001 |
|  | 70-79 | 0.68±0.08 | 0.82±0.13 | ＜0.0001 |
|  | ≥80 | 0.71±0.07 | 0.89±0.09 | ＜0.0001 |
| The tortuosity of AA | <40 | 0.61±0.11 | 0.68±0.16 | 0.157 |
|  | 40-49 | 0.57±0.06 | 0.65±0.05 | ＜0.0001 |
|  | 50-59 | 0.59±0.04 | 0.65±0.05 | 0.001 |
|  | 60-69 | 0.59±0.04 | 0.69±0.08 | ＜0.0001 |
|  | 70-79 | 0.61±0.08 | 0.72±0.10 | 0.003 |
|  | ≥80 | 0.60±0.03 | 0.75±0.10 | ＜0.0001 |

DTA: descending thoracic aorta; AA: abdominal aorta

**Supplementary Table 3 Characteristics of participants in the published article.**

| Variables | Male (n=592, 64.9%) | Female (n=320, 35.1%) | *p* value |
| --- | --- | --- | --- |
| Age, years | 50.5±9.8 | 52.4±9.0 | ＜0.01 |
| BMI, kg/m^2^ | 23.7±3.1 | 22.1±2.9 | ＜0.001 |
| Current smoking, n (%) | 180 (30.4%) | 17 (5.3%) | ＜0.001 |
| SBP, mmHg | 122.9±14.4 | 115.3±14.7 | ＜0.001 |
| DBP, mmHg | 78.7±9.3 | 71.4±9.5 | ＜0.001 |
| Glucose, mg/dL | 99.7±11.5 | 94.9±17.4 | ＜0.001 |
| Cholesterol, mmol/L | 207.1±34.8 | 214.9±37.6 | ＜0.001 |
| Triglycerides, mmol/L | 115.7±82.3 | 70.6±46.6 | ＜0.001 |
| baPWV, cm/s | 1411.5±241.1 | 1363.0±241.3 | 0.10 |
| eGFR, mL/min/1.73cm^2^ | 69.3±11.6 | 72.5±12.5 | ＜0.001 |

**Supplementary Table 4 The Intraclass Correlation of Intra‐ and** **interobserver reproducibility for the assessment of aortic diameters and tortuosity index**

| Variables | Intraobserver | | Interobserver | |
| --- | --- | --- | --- | --- |
|  | ICC | 95% CI | ICC | 95% CI |
| L1, mm | 0.926 | 0.844 to 0.965 | 0.847 | 0.669 to 0.928 |
| L2, mm | 0.987 | 0.972 to 0.994 | 0.920 | 0.84 to 0.961 |
| L3, mm | 0.989 | 0.976 to 0.995 | 0.921 | 0.84 to 0.961 |
| L4, mm | 0.994 | 0.988 to 0.997 | 0.922 | 0.844 to 0.962 |
| L5, mm | 0.962 | 0.923 to 0.982 | 0.894 | 0.79 to 0.948 |
| Aorta tortuosity | 0.974 | 0.944 to 0.987 | 0.865 | 0.736 to 0.934 |
| DTA tortuosity | 0.913 | 0.827 to 0.958 | 0.923 | 0.78 to 0.968 |
| AA tortuosity | 0.875 | 0.756 to 0.938 | 0.893 | 0.778 to 0.949 |

CI: Confidence Interval; DTA: Descending thoracic aorta; AA: abdominal aorta;
