# Supplementary material for: The sex-specific difference in age-related aortic regional morphological changes
Source: Aging Clin Exp Res. 2025 Mar 11;37(1):76. doi: 10.1007/s40520-025-02981-1 (PMC11897083; doi:10.1007/s40520-025-02981-1)
Supplement: Supplementary file 2 — Supplementary file2 (PPTX 506 KB) [file 40520_2025_2981_MOESM2_ESM.pptx]

## Slide 1
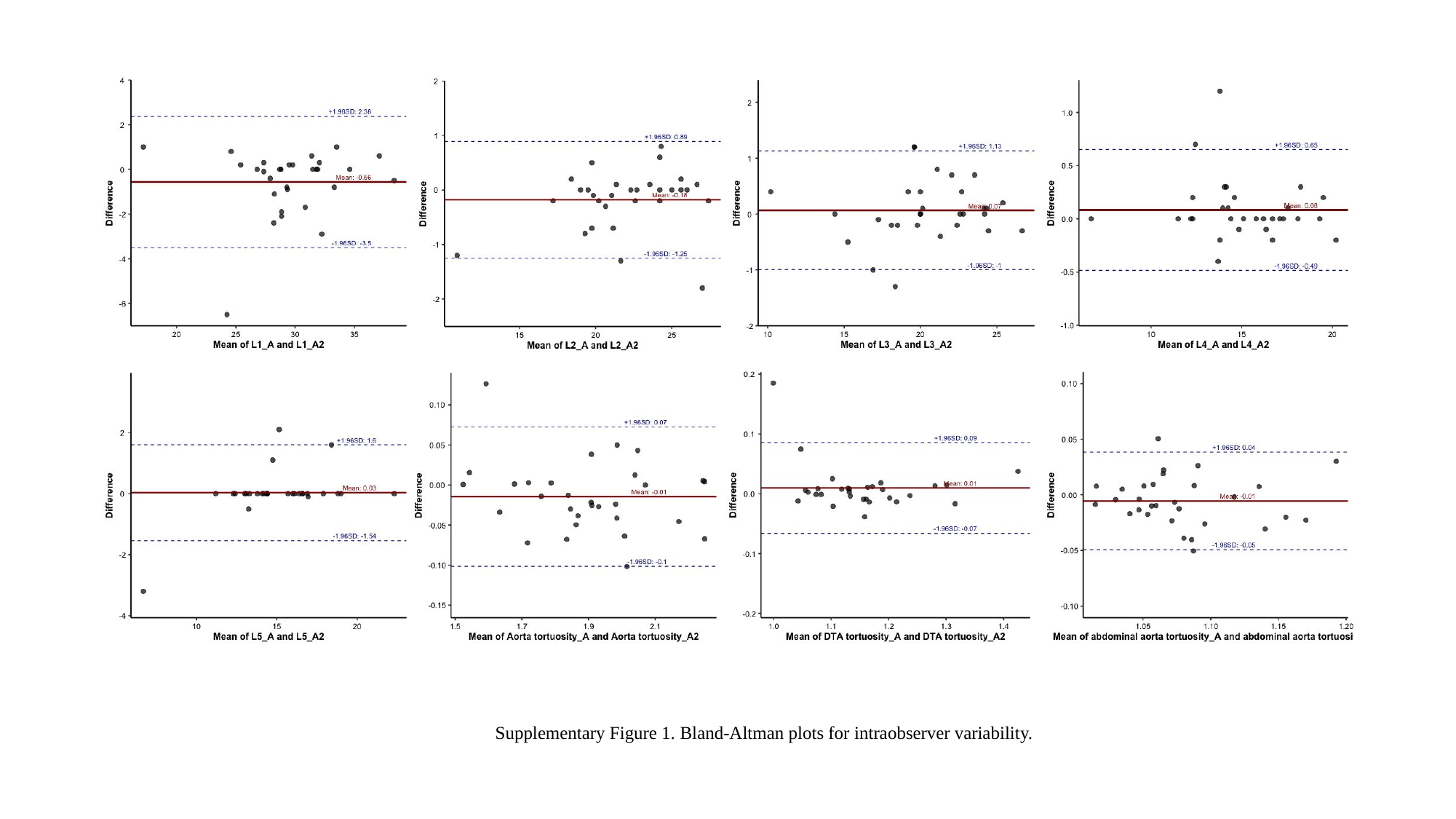

Supplementary Figure 1. Bland-Altman plots for intraobserver variability.

## Slide 2
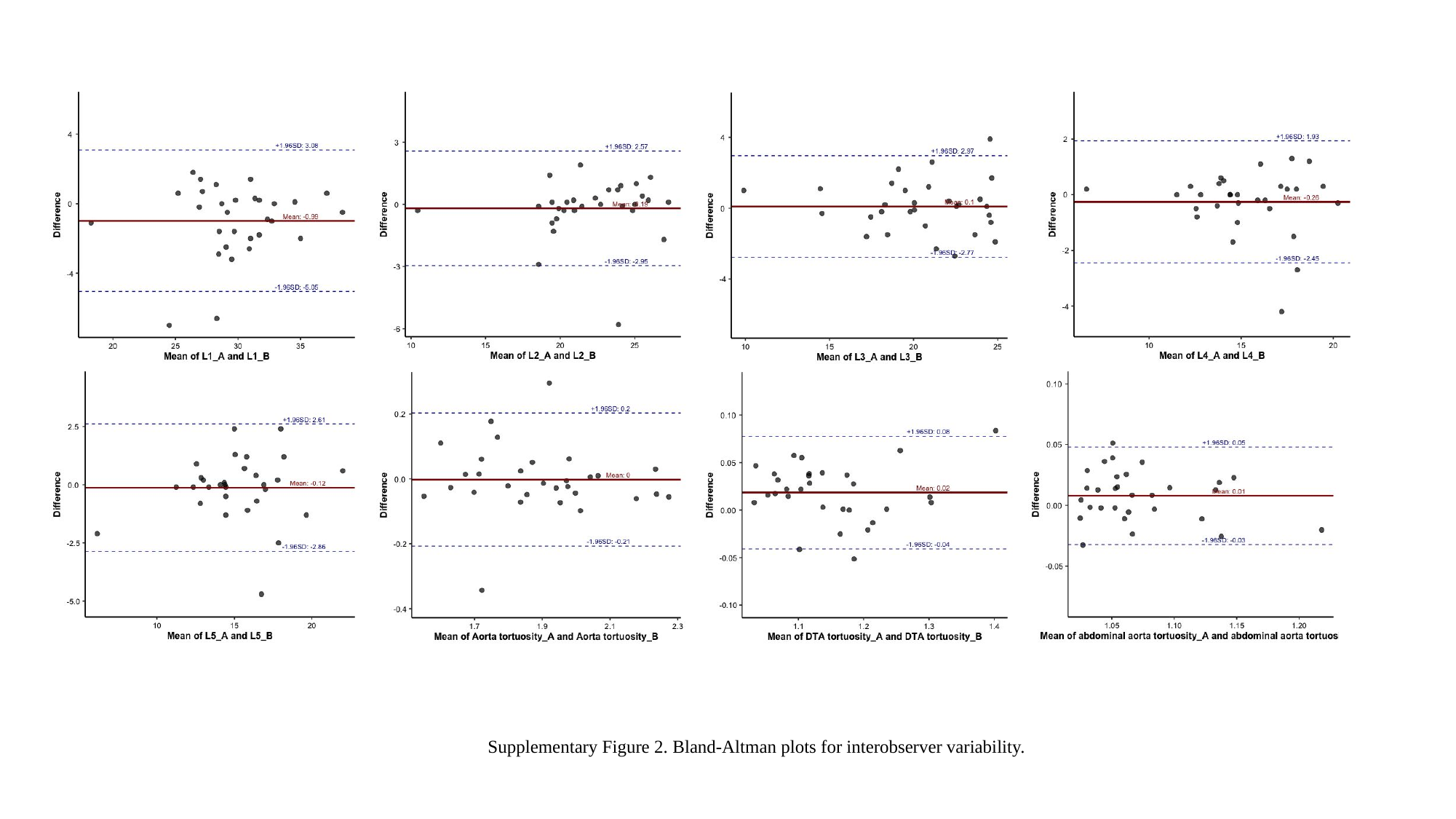

Supplementary Figure 2. Bland-Altman plots for interobserver variability.
